# Are cefiderocol or sulbactam/durlobactam better than alternative best available treatment for infection by carbapenem-resistant *A. baumannii*? A systematic literature review

**DOI:** 10.1007/s15010-025-02527-7

**Published:** 2025-04-07

**Authors:** Stamatis Karakonstantis, Petros Ioannou, Diamantis P. Kofteridis

**Affiliations:** 1 Department of Internal Medicine and Infectious Diseases, University Hospital of Heraklion, Crete, Greece; 2 Faculty of Medicine, University of Crete, Heraklion, Greece

**Keywords:** Acinetobacter baumannii, Cefiderocol, Sulbactam/durlobactam

## Abstract

**Purpose:**

Cefiderocol (CFDC) and sulbactam/durlobactam (SUL/DUR) are new treatment options against infections by carbapenem-resistant *A. baumannii* (CRAB). However, whether they outperform contemporary alternative best available therapy (BAT), currently consisting of high-dose ampicillin/sulbactam (AMP/SUL)-based regimens, is unclear.

**Methods:**

A systematic review was conducted in PubMed and clinical trial registries to assess regimens used in comparator arms in studies comparing CFDC or SUL/DUR to alternative treatment regimens.

**Results:**

Only 1 relevant study was found for SUL/DUR (the registrational Phase 3). Almost all (98%) patients enrolled had pneumonia and the comparator arm was colistin/imipenem, a regimen not recommended for treatment of CRAB infections, especially pneumonia. With regards to CFDC, subgroup analyses (with significant limitations) from 2 randomized trials were disappointing showing higher mortality in CREDIBLE-CR compared to colistin-based treatment and similar mortality in APEKS-NK compared to high-dose meropenem among patients with CRAB infections. The rest (*n* = 11) of the trials were observational, predominantly single-center (82%) and retrospective (82%), and all but one were conducted in Italy (91%). Although meta-analyses of observational studies suggest better outcomes with CFDC, the comparator arm was colistin-based in all cases and only a minority of patients had received high-dose AMP/SUL.

**Conclusion:**

High-quality evidence supporting use of either CFDC or SUL/DUR in favor of high-dose AMP/SUL-based regimens is lacking. This has important stewardship implications. Additionally, both CFDC and SUL/DUR are much more expensive than AMP/SUL, an important consideration especially for low-/mid-income countries. Studies comparing CFDC- and SUL/DUR-based treatments to contemporary alternative BAT are needed.

## Introduction

Carbapenem-resistant *A. baumannii* (CRAB) with resistance to last resort antibiotics (including polymyxins, ampicillin/sulbactam, tigecycline and aminoglycosides) is increasingly reported globally [1] and is associated with considerable excess mortality [2, 3] and limited treatment options [4, 5], often based on synergistic combinations supported by low-quality clinical evidence [6–9]. Fortunately, two new β-lactam antibiotics, cefiderocol (CFDC) and, more recently, sulbactam/durlobactam (SUL/DUR), are available to combat these pathogens and may improve outcomes compared to alternative, predominantly colistin(CST)-based, treatment options [10–13].

According to the latest guidelines by IDSA the currently preferred treatment option for infections by CRAB is SUL/DUR combined with a carbapenem [14]. In contrast, IDSA suggests the use of CFDC as a last resort option due to; (a) challenges in susceptibility testing, (b) inferior efficacy against CRAB in a post-hoc analysis of CREDIBLE-CR [10], and (c) limitations in available observational studies [14]. Nevertheless, above-mentioned subgroup analysis has major limitations due to imbalances in patient characteristics between treatment groups [12, 13]. Furthermore, subsequent observational studies and meta-analyses suggest better outcomes with CFDC-based regimens [11–13, 15].

However, there is concern that we may already be losing CFDC against *A. baumannii* (especially against NDM-producing strains) [16–18], while SUL/DUR lacks activity against metallo-β-lactamase-producing strains [19]. Therefore, antimicrobial stewardship is essential to prevent resistance development. Additionally, these agents are significantly more expensive than alternative options.

Given these considerations, it is critical to evaluate whether these new options (CFDC and SUL/DUR) provide superior outcomes compared to alternative best-available therapy (BAT), which, per current IDSA guidance, consists of high-dose ampicillin/sulbactam (AMP/SUL) combined with another agent [14]. Notably, AMP/SUL-based regimens were used in only a minority of patients in CFDC’s registrational trial for CRAB infections [10], while the comparator arm in the SUL/DUR registrational trial consisted of CST plus imipenem [20].

The aim of this systematic review is to analyze the comparator regimens used in studies assessing CFDC or SUL/DUR against alternative treatments.

## Methods

The following search was conducted in PubMed from inception to 29 November 2024; (cefiderocol OR durlobactam) AND (Acinetobacter OR baumannii). Additionally, searches were performed in CENTRAL, ClinicalTrials.gov and WHO International Clinical Trials Registry Platform. Studies were eligible for review if they compared outcomes of treatment with CFDC or SUL/DUR against alternative options in patients with CRAB infections.

The goal was to assess the comparator antibiotic regimens used in each trial, and determine whether these reflect BAT. BAT was defined as treatment regimens including AMP/SUL, particularly high-dose AMP/SUL for isolates resistant to AMP/SUL based on CLSI breakpoints [21], in accordance with existing guidelines [14].

## Results

### Summary of included studies

Of the *n* = 371 articles retrieved, *n* = 14 eligible studies were reviewed [10, 20, 22–33]; *n* = 13 comparing CFDC-based to alternative regimens [10, 22–33] and *n* = 1 comparing SUL/DUR to CST (both arms in combination with imipenem) [20]. The included studies, along with their characteristics and findings, are summarized in Tables 1 and 2.

**Table 1:** Summary of studies

Study	Study description	CFDC or SUL/DUR regimens	Comparator regimen(s)	Sites of infection- Total patients (CFDC vs. other arm)	Notes
Buonomo AR, 2024 [22]	*Design*; Observational, prospective *Population*; Adults with CRAB infection. *Participating centers*; University of Naples Federico II, Italy. *Study period*; 1 January 2022 to 31 August 2023	Total *n* = 18 CFDC monotherapy 61% CFDC combination + AMP/SUL 28% + FOS 5.5% + TGC 5.5% CFDC 2gr q8h	Total *n* = 27 CST used in combination with other agents in all patients; + AMP/SUL 82% + FOS 11% + TGC 7% CST 9 million IU loading dose followed by 4.5 million IU q12h, TGC 100 mg loading dose followed by 50 mg q12h, FOS 12–24 g/day, AMP/SUL 27 g/day	VAP 42% HAP 4% CVC BSI 31% IAI 7% UTI 4% SSTI 16% Endocarditis 2% Osteomyelitis 4% Device infection 2%	CFDC treatment was associated with significantly higher early clinical success (100% vs. 63%) and higher overall clinical success, albeit non-significantly (72% vs. 63%). AMP/SUL was not significantly associated with either early or overall clinical success.
Oliva A, 2024 [23]	*Design*; Observational, retrospective *Population*; Adults ≥ 18 with CRAB BSI treated with either CFDC or CST. *Participating centers*; Sapienza University of Rome, Italy *Study period*; June 2021 to April 2023	CFDC monotherapy 28% Combination; + AMP/SUL 48% + FOS 24% + TGC 12% + MEM 2% CFDC 2 gr loading dose followed by a 3 h infusion of 2 g q8h	CST monotherapy 22% Combination; + FOS 37% + AMP/SUL 33% + TGC 11% + MEM 13% CST 9 million IU loading dose followed by 4.5 million IU q12h; AMP/SUL total daily dose of 24–27 g (4 g/2 g q6h or 6 g/3 g q8h); FOS total daily dose of 16–18 g, divided q6–8 h; TGC first dose 100–200 mg, followed by 50–100 mg q12h; MEM 2 g loading dose followed by 2 g q8h.	Primary BSI 43% (36% vs. 50%) CVC BSI 20% (26% vs. 15%) VAP BSI 26% (28% vs. 24%) HAP BSI 10% (8% vs. 11%)	Mortality was lower in CFDC group at 14 days (22% vs. 31%, *p* = 0.276), 30 days (36% v 42%, *p* = 0.682) and overall (57% vs. 61%, *p* = 0.682), albeit non-significantly Length of hospital and ICU stays was longer in CFDC arm. Clinical cure was significantly higher in CFDC arm (66% vs. 44%, *p* = 0.027). Benefit of CFDC in mortality and clinical cure was more pronounced in patients with bacteremic HAP/VAP. CFDC was not associated with mortality in the multivariable analysis.
Rodríguez-Aguirregabiria M, 2024 [33]	*Design*; Observational, retrospective. *Population*; Adults (≥ 18) ICU patients with CRAB infections. *Participating centers*; Hospital Universitario La Paz, Madrid, Spain. *Study period*; October 2022 to November 2023	*n* = 13 CFDC monotherapy 62% CFDC combination; + CST 15% + CST/LIN 8% + LIN 8% + CST/AMP/SUL 8%	*n* = 6 CST monotherapy 17% CST combination; + AMP/SUL 33% + MEM 17% + TZP 17% + LIN 17% Doses are not reported.	VAP 53% (54% vs. 50%) BSI 47% (46% vs. 50%)	Mortality was 23% (3/13) among CFDC-treated patients vs. 50% (3/6) among CST-treated patients.
Russo A, 2024 [24]	*Design*; Observational, retrospective. *Population*; Adults ≥ 18 with severe CRAB infections treated with FOS-based regimens. *Participating centers*; 4 hospitals in Italy: (1) ‘Mater Domini’ Hospital of ‘Magna Graecia’ University of Catanzaro, (2) Cristo Re Hospital, Rome, (3) Sapienza University of Rome, (4) Cotugno-Monaldi Hospital, Naples. *Study period*; December 2017 to December 2022.	CFDC + FOS (*n* = 54) Some patients received > 2 antibiotics but the exact combinations are not reported. Dosing regimens are not reported.	FOS + CST (*n* = 48) FOS + AMP/SUL (*n* = 18) FOS + MEM (*n* = 8) FOS + TGC (*n* = 18) FOS + SXT (*n* = 2) Some patients received > 2 antibiotics but the exact combinations are not reported. Dosing regimens are not reported.	VAP 59% Primary BSI 22% CVC BSI 16% Other 4%	CST-based treatment was associated with a higher hazard of 30-day mortality, while CFDC-based treatment was associated with lower mortality. 30-day mortality per treatment regimens; CFDC + FOS; 37% FOS + MEM; 50% FOS + CST; 63% FOS + TGC; 22% FOS + AMP/SUL; 67%
Bavaro DF, 2023 [25]	*Design*: Observational, retrospective *Population*: Adults ≥ 18 with monomicrobial BSI by CRAB being treated with CFDC- or CST-based regimens for at least 48 h. Patients with concurrent COVID-19 or co-infection at other site were excluded. Patients that died within 48 h or discontinued treatment within 48 h were also excluded. *Participating centers*: Single-center. University hospital “Policlinico”, Bari, Italy. *Study period*; 1 January 2020- 31 December 2022	CFDC-based; monotherapy 37% Combination 63%; + AMP/SUL 23% + FOS 47% Dosing regimens not reported.	CST-based; monotherapy 4% Combination 96%; + AMP/SUL 33% + FOS 29% + TGC 49% + >2 drugs 64% Dosing regimens not reported.	BSI sites; Primary /UTI 37% (37% vs. 28%) CVC-related 31% (26% vs. 33%) IAI 16% (7% vs. 21%) Lung 10% (16% vs. 7%) SSTI 10% (14% vs. 8%) Endovascular 1% (0% vs. 1%) Osteoarticular 1% (0% vs. 1%)	Detailed data on outcomes of patients treated with high-dose AMP/SUL are not available. Nevertheless, unadjusted 30-day all-cause mortality HR for different treatment regimens are reported as follows; CFDC-based; 0.51 (95% CI 0.28–0.94) SUL-based; 0.98 (95% CI 0.66–1.77) FOS-based; 0.43 (95% CI 0.22–0.81) TGC-based; 1.65 (95% CI 0.95–2.85)
Dalfino L, 2023 [26]	*Design*: Prospective, observational *Population*: Adults ≥ 18 in a non-COVID-19 ICU with monomicrobial VAP due to CRAB with at least one active in vitro antibiotic. Patients with duration of active treatment less than 72 h were excluded. *Participating centers*: Single-center. University hospital “Policlinico”, Bari, Italy. *Study period*; 11 March 2021 to 31 December 2022.	CFDC 2 g q8h infused over 3 h. 47.5% alone, 52.5% in combination with FOS. All patients also received inhaled CST 2 million IU q8h	CST dosed as recommended by guidelines [56] All in combination with a 2nd antimicrobial; + TGC 54% (200 mg loading dose followed by 100 mg q12h) + high-dose AMP/SUL 30% + MEM 28% All patients also received inhaled CST 2 million IU q8h	VAP Bacteremic VAP 32%	Unudjusted clinical resolution; CFDC alone 53% CFDC + FOS 95% CST alone 52% CST + TGC 41% CST + AMP/SUL 53% CST + MEM 0% Other outcomes are not reported separately for each treatment regimen.
Kaye KS, 2023 [20]	*Design*: RCT *Population*: Adults ≥ 18 with HAP/VAP/BSI by ABC. APACHE II score 10–30, SOFA score < 12. Efficacy analyses conducted in patient with CRAB susceptible to CST and SUL/DUR. *Participating centers*: 16 countries (North America, South America, Europe, and Asia), 59 clinical sites	SUL/DUL (1/1 g q6h infused over 3 h) + IMI (1gr q6h infused over 1 h)	CST (loading dose 75000–150000 IU/kg followed by 75000 IU/kg) + IMI (1gr q6h infused over 1 h)	HAP/VAP 98% (100% vs. 0%) BSI 2% (0% vs. 2%)	SUL/DOR was associated with lower 28-day mortality (difference − 13·2%, 95% CI -30 to 3·5). Secondary outcomes also favored sulbactam-durlobactam, including clinical cure (62% versus 40%), microbiologic response (68% versus 42%) at the test of cure visit, and a lower risk of nephrotoxicity (13% versus 38%).
Mazzitelli M, 2023 [27]	*Design*; Observational, retrospective *Population*; Adults with infection by CRAB treated with CFDC or CST. *Participating centers*; Single-center. Padua University Hospital, Italy. *Study period*; August 2021 to July 2022 for CFDC-treated patients. August 2020-July 2021 for CST-treated patients.	CFDC monotherapy 50% Combination; + FOS 13% + MEM 22% + TGC 30% Dosing regimens are not reported.	CST combination therapy in all patients; + TGC 96% + MEM 80% + 6% FOS Dosing regimens are not reported.	BSI 48% (57% vs. 37%) Pneumonia 52% (43% vs. 63%)	No significant difference in mortality comparing CFDC to CST (51% vs. 37%), clinical cure (73% vs. 67%) or microbiological cure (43% vs. 41%). Acute kidney injury was significantly more common in CST group.
Rando E, 2023 [28]	*Design*; Observational. Semi-retrospective design. A prospective registry was formulated following availability of CFDC in April 2021. *Population*; COVID-19 ICU patients with VAP by CRAB being treated with CRAB-targeted therapy within 24 h of diagnosis. *Participating centers*; Fondazione Policlinico Universitario Agostino Gemelli IRCCS, Columbus COVID II Hospital, Rome, Italy; *Study period*; September 2020 to December 2022	CFDC monotherapy 22% CFDC combination; + CST 29% + CST/TGC 20% + CST/FOS 7% + TGC 5.5% + FOS 5.5% + TGC/CST/FOS 4% + TGC/FOS 2% + AMP/SUL 2% + CST/AMP/SUL 2% + CST/MEM/TGC 2% CFDC 2 g as an extended infusion of 3 h q8h. High-dose nebulized colistimethate (5 MIU in 6 mL of normal saline solution over 30 min every 8 h) was used in all patients.	CST monotherapy 1.5% CST combination; + TGC/FOS 73% + MEM/TGC 12% + TGC 11% + FOS 1.5% + MEM/TGC/FOS 1.5% CST 9 million IU loading dose and then 4.5 million IU q12h in 2-hour infusions, FOS 8 g q8h in 2-hour infusions, TGC 200 mg as a loading dose, and then 100 mg q12h in 1-hour infusions and AMP/SUL 9 g q8h in 4-hour infusions. High-dose nebulized colistimethate (5 MIU in 6 mL of normal saline solution over 30 min every 8 h) was used in all patients.	VAP 100% with BSI 17% (15% vs. 18%)	CFDC was associated with lower 28-day mortality (44% vs. 66%, *p* = 0.011). CFDC was independently associated with lower mortality in propensity-adjusted multiple logistic regression.
Russo A, 2023 [29]	*Design*; Observational, retrospective *Population*; Adults ≥ 18 in a COVID-19 ICU with monomicrobial bacteremic VAP due to CRAB. *Participating centers*; Single-center. ‘Mater Domini’ Hospital of ‘Magna Graecia’ University of Catanzaro in Italy. *Study period*; March 2020 to August 2022.	CFDC 2gr q8h. 100% in combination regimens; + FOS 31.5% + FOS + TGC 15.8% + FOS + TGC + MEM 15.8% + SXT 10.5% + TGC 5.2% + AMP/SUL 5.2% + MEM + FOS + TGC + SXT 5.2%	CST monotherapy 22.2% CST combination 88% + MEM + TGC 22% + MEM 16.6% + TGC 11.1% + FOS 5.5% + SXT 5.5% + SXT + MEM + TGC 6% + MEM + FOS 4% + MEM + TGX + AMP/SUL 4% + SXT + TGC 4% CST loading dose 9 million IU followed by 4.5 q12h. TGC loading dose 100 mg followed by 5 mg q12h GEN 5 mg/kg q24h RIF 10 mg/kg/day MEM 2gr q8h or 1.5 g q6h FOS loading dose 8 g followed by 12–24 g/day dosed q6-8 h AMP/SUL 3gr q6h SXT 15-20 mg/kg/d divided q6h CST aerosol 2 million IU q8h	Bacteremic VAP	Note that dosing regimens of TGC and AMP/SUL are lower than the recommended for CRAB. Also note the large proportion of patients in the CFDC arm receiving FOS. Considerably lower 14-day and 30-day mortality comparing CFDC- to CST-based treatment (5% vs. 76% and 32% vs. 98% respectively). CFDC-containing and CFDC + FOS were independently associated with lower mortality.
Falcone M, 2022 [30]	*Design*; Observational, retrospective. *Population*; Adults ≥ 18 with CRAB infections treated with CFDC- or CST-based regimens for at least 48 h. Polymicrobial infection involving Gram-positive bacteria, fungi, or Gram-negative bacteria resistant to CST or CFDC were excluded. *Participating centers*; University hospital of Pisa, Italy. *Study period*; January 2020 to August 2021.	CFDC monotherapy 32% CFDC combination; + TGC 44% + FOS 17% + ETP 2% + AMP/SUL 2% + MEM/VAB 2% CFDC 2gr q8h infused over 3 h	CST monotherapy 17% CST combination; + TGC 51% + TGC/MEM 9% + TGC/RIF 4% + TGC/FOS 4% + MEM/FOS 3% + RIF 1% + aminoglycoside 1% + AMP/SUL 1% + TGC/SAM 8% Usual dosing regimens as follows; CST, loading dose of 9 million IU followed by 4.5 million IU q12h; TGC loading dose of 200 mg followed by 100 mg q12h; GEN 5 mg/kg q24h; FOS 12–24 g/day divided q6–8 h; AMP/SUL 3 g every 6 h; MEM 1–2 g q8 h in extended infusion	BSI 64% (57% vs. 68%) VAP 29% (26% vs. 30%), Other 8% (17% vs. 3%)	30-day mortality was higher in patients treated with CST-based vs. CFDC-based regimens (55.8% vs. 34%, *p* = 0.018). CFDC was independently associated with lower mortality in multivariable analysis. Nephrotoxicity was more common CST group. Microbiological failure was lower in CFDC group (6.8% vs. 17.4%, *p* = 0.079).
Bassetti M, 2021 [10]	*Design*: RCT *Population*: Adults ≥ 18 with HAP/VAP/HCAP/BSI/cUTI with evidence of CR-GNB. *Participating centers*: 95 hospitals in 16 countries (North America, South America, Europe, and Asia)	CFDC 2 g q8h infused over 3 h Monotherapy (83%)* Combination therapy (18%)*; + TGC + FOS (3%) + AMK (1%) + AMP/SUL (1%) + CIP (1%) + GEN (1%) + GEN, TGC (1%) + LVX (1%) + TZP (1%)	CST-based (66%)* CST alone 16% CST combination 50% AMP/SUL-based regimens (unclear dose); CST + AMP/SUL 5% (*n* = 2) PMB + AMP/SUL + FOS 3% (*n* = 1). Assuming that above treatment regimens were uses in CRAB infections this corresponds to 18% of CRAB patients in the BAT arm.	CRAB subgroup; PN 70% vs. 59%, BSI 27% vs. 41%, cUTI 3% vs. 0%	Mortality was higher (albeit non-significantly) in CDFC group, even after adjusting for covariates (HR for mortality of 1.53 [95% CI 0.35, 6.71) [12]]
Pascale R, 2021 [31]	*Design*: Observational, retrospective. *Population*: Adult (≥ 18) ICU patients with CRAB infection. *Participating centers*: 3 hospitals in Bologna, Italy. IRCCS Policlinico di Sant’Orsola, Bellaria Hospital and Maggiore Hospital *Study period*: January 2020 o April 2021	CFDC 2gr q8h Monotherapy in all cases.	CST monotherapy 18% CST combinations; + MEM 41% + SXT 14% + TGC 15% + AMP/SUL 12% + FOS 7%	LRTI 41% (33% vs. 46%) BSI 58% (64% vs. 54%) CVC-BSI 13% (7% vs. 17%) LRTI-BSI 50% (63% vs. 40%)	There was no significant difference in any outcome (14-day death, AKI, clinical cure at 14 days, microbiological cure at 14 days, 28-day mortality). In multivariable analysis CFDC was associated with a lower hazard of 28-day mortality, albeit non-significantly (HR 0.64, 95% CI 0.38–1.08)
Wunderink RG, 2021 [32]	*Design*: RCT *Population*: Adults ≥ 18 with HAP/VAP/HCAP by GNB *Participating centers*: 17 countries (Asia, Europe, USA), 76 centers	CFDC 2 g q8h infused over 3 h + Linezolid 600 mg q12h	MEM 2 g q8h infused over 3 h + Linezolid 600 mg q12h	HAP/VAP/HCAP	CFDC was not better than MEM even among isolates with MEM MIC > 8 mg/L

ABC = Acinetobacter baumannii complex, AKI = acute kidney injury, CRAB = carbapenem-resistant *A. baumannii*, CR-GNB = carbapenem-resistant Gram-negative bacteria, CST = colistin, cUTI = complicated urinary tract infection, CVC = central venous catheter, DUR = durlobactam, ETP = ertapenem, FOS = fosfomycin, GEN = gentamicin, GNB = Gram-negative bacteria, HAP = hospital-acquired pneumonia, HCAP = healthcare-associated pneumonia, IAI = intra-abdominal infection, ICU = intensive care unit, IMI = imipenem, IU = international units, LIN = linezolid, LRTI = lower respiratory tract infection, MEM = meropenem, MIC = minimum inhibitory concentration, PN = pneumonia, RIF = rifampicin, RCT = randomized controlled trial, SSTI = skin and soft tissue infection, SUL = sulbactam, SXT = trimethoprim/sulfamethoxazole, TGC = tigecycline, UTI = urinary tract infection, VAB = vaborbactam, VAP = ventilator-associated pneumonia

* carbapenem-resistant microbiological intention-to-treat population

**Table 2:** Summary of study characteristics

	CFDC vs. other	SUL/DUR vs. Other
Total number of studies	13	1
RCTs	2	1
Observational studies	11	0
Retrospective	9 (82%)	NA
Single-center	9 (82%)**	NA
Conducted in Italy	10 (91%)*	NA
Sites of infection (% of all patients)		
PN	**10%** [25], **29%** [30], **36%** [23], **41%** [31], **46%** [22], **52%** [27], **53%** [33], **59%** [24], **100%** [26, 28, 29]	98%
BSI (excluding bacteremic PN)	**17%** [28], **31%** [22], **32%** [10, 26], **38%** [24], **47%** [33], **48%** [27], **58%** [31], 64% [30], **100%** [23, 25, 29]	2%
% of patients in comparator arm being treated with an AMP/SUL-based regimen	**0%** [27, 28, 32], **4%** [29]^#^, **9%** [30]^#^, **12%** [31], **18%** [10], **30%** [26], **33%** [23, 25, 33], **38%** [24], **82%** [22]	0%
% of patients in the CFDC or SUL/DUR arm receiving combination therapy	**0%** [31, 32], **18%** [10], **37%** [25], **38%** [33], **49%** [22], **50%** [27], **52%** [26], **72%** [23], **78%** [28], **100%** [29]	100% combined with imipenem
% of patient receiving combination of CFDC with FOS	**0%** [31, 32], **3%** [10], **5%** [22], **13%** [27, 30], **17%** [30], **18%** [28], **24%** [23], **53%** [26], **47%** [25], **68%** [29], **100%** [24]	NA

CFDC = cefiderocol, SUL/DUR = sulbactam/durolobactam

* Two of the studies were conducted in the same hospital and overlapping period, one reporting on cases with ventilator-associated pneumonia [26], the other reporting on patients with bloodstream infections [25]

** Two hospitals have published studies both separately [23, 29] and as part of a multicenter study [24]

^#^ In 2 studies [29, 30], a low-dose regimen of AMP/SUL was used

Pneumonia was the most frequently reported site of infection across studies. Aside from the 3 randomized trials discussed in the next paragraph, the remaining 11 studies were observational, mostly retrospective (82%) and single-center (82%), with nearly all (91%) conducted in Italy (91%). All observational studies compared CFDC-based with CST-based therapy. Treatment regimens in both groups were highly heterogeneous, including both monotherapy and combination with various antimicrobials.

### Overview of the 3 randomized trials

Three eligible randomized controlled trials were reviewed [10, 20, 32]. In the registrational Phase 3 trial of SUL/DUL the comparator was CST (both arms combined with imipenem/cilastatin) [20]. Notably, almost all patients (98%) had pneumonia [20]. In CREDIBLE-CR [10], one of the registrational trials of CFDC, CFDC was compared to “BAT”. However, detailed data on treatment regimens used in the subgroup of patients with CRAB infection were not provided, though most study patients overall received CST-based treatment (Table 1). A sulbactam-based regimen was used in only a minority of patients in “BAT” arm. Among patients with CRAB infection, pneumonia was the most common site of infection (70% in CFDC arm vs. 59% in “BAT” arm), followed by bacteremia (27% in CFDC arm vs. 41% in “BAT” arm). In APEKS-PN, another registrational trial comparing CFDC with high-dose extended infusion meropenem for pneumonia by Gram-negative pathogens, CFDC unexpectedly failed to outperform meropenem in a subgroup analysis of patients infected by carbapenem-resistant *A. baumannii* [32].

### Observational studies on CFDC- vs. CST-based treatment

CFDC was used either as monotherapy or more commonly in combination with various other antimicrobials, notably fosfomycin, AMP/SUL or tigecycline (Tables 1 and 2). The majority of comparator regimens were based on CST, predominantly combined (78–100%) with various other antimicrobials (Table 1).

In most studies, only a minority of patients in the comparator group were treated with a regimen containing AMP/SUL (0% [20, 27, 28, 32], 4% [29], 9% [30], 12% [31], 18% [10], 30% [26], 33% [23, 25, 33], 38% [24], 82% [22]), predominantly in combination with CST (Table 1). Dosing regimens for non-CFDC antibiotics used were not reported in several of the studies [10, 24, 25, 27, 31, 33]. A high-dose AMP/SUL regimen was reported in three of the studies [22, 23, 26], while in two studies a low-dose regimen was reported [29, 30].

A comparison of outcomes in patients treated with AMP/SUL-based regimens compared to CFDC-based regimens was available in few studies, with small number of patients in both CFDC and AMP/SUL arms. In one study clinical resolution was similar (53%) comparing CFDC + inhaled CST to CST + AMP/SUL + inhaled CST, but higher (95%) in patients treated with CFDC + fosfomycin + inhaled CST [26]. In another study from the same hospital, CFDC-based but not SUL-based treatment was associated with lower unadjusted hazard for mortality [25]. Of note the majority of patients in that study received CFDC in combination with FOS (47% vs. 29% in CST arm) or AMP/SUL (23% vs. 33% in CST-arm), and fosfomycin was independently associated with lower hazard of mortality [25]. In a multi-center study in Italy mortality was significantly higher in patients treated with a fosfomycin + AMP/SUL regimen (*n* = 18), compared to patients treated with CFDC + fosfomcycin (*n* = 54) (67% vs. 37%) [24].

Of interest in most studies showing benefit of CFDC-based regimens, a large proportion of patients received CFDC in combination with fosfomycin (13% [30], 18% [28], 53% [26], 47% [25], 68% [29], 100% [24]), and use of fosfomycin was independently associated with lower mortality in 3 of these studies [24, 25, 29]. In 2 other studies showing statistically significant reduction in mortality by CFDC-containing regimens CFDC was most often combined with TGC (55% [30], 31% [28]) or FOS (13% [30], 18% [28])

## Discussion

Our systematic review highlights the lack of clinical evidence showing that either CFDC or SUL/DUR can outperform alternative BAT, defined as a high-dose AMP/SUL-based regimen. Especially in the case of SUL/DUR, the comparator (CST/imipenem) used in the registrational trial is far from optimal [20]. CST has significant limitations [14], including poor penetration into the epithelial lining fluid, a crucial factor given that nearly all enrolled patients had pneumonia [20]. Additionally, randomized trials and meta-analyses have shown that adding a carbapenem to CST is unlikely to provide any benefit [14, 34–37] and this combination is not recommended [14]. In short, the comparator used in the SUL/DUR registrational trial does not reflect a regimen that any contemporary infectious diseases clinician would prescribe. Regarding CFDC, the comparator regimens were highly heterogeneous and, in most cases, CST-based, a significant limitation as the majority of patients in relevant studies had pneumonia. Importantly, a high-dose AMP/SUL-based regimen was only used in a minority of patients.

Even in studies reporting CFDC or SUL/DUR use as rescue therapy after failure of other treatment regimens, prior treatments were either inappropriate or suboptimal, most commonly CST-based [38–44]. Notably, in one case SUL/DUR was used as rescue therapy for an infection by an isolate with sulbactam MIC 8 mg/L (suggesting that the much cheaper and widely available AMP/SUL could be a suitable option as well) [38]. In another case [45], SUL/DUR was successfully used as salvage therapy in a patient with meningitis following failure of the combination of CFDC with AMP/SUL (though the latter was sub-optimally dosed), suggesting potential benefit in selected patients. Importantly, we did not encounter any study describing salvage therapy with CFDC or SUL/DUR after failure of an appropriate high-dose AMP/SUL-based regimen.

Regarding CFDC, concern remains about whether it outperforms alternative BAT. Warning signals about the efficacy of CFDC against CRAB infections first emerged from the CREDIBLE-CR Phase 3 trial [10]. However, as highlighted by subsequent publications [12, 13], this was based on a subgroup analysis with considerable bias and limitations. Subsequent meta-analyses based on observational data suggest a lower-risk of mortality and acute kidney injury comparing CFDC- vs. non-CFDC (predominantly CST)-based regimens [11–13, 15], although CFDC was not significantly better in other endpoints (clinical cure rate, microbiological cure rate, duration of hospitalization), based on few studies and small number of patients [11, 15]. Interestingly, in APEKS-NP, a phase 3 trial comparing CFDC to high-dose, extended-infusion meropenem in patients with nosocomial pneumonia, CFDC was not better in the subgroup of patients with pneumonia by CRAB, even though meropenem lacks activity against CRAB [32], a potential explanation being the problematic differentiation of infection from colonization. It is also important to note that, similar to CST (and unlike SUL and DUR [46]), CFDC also has suboptimal penetration in the epithelial lining fluid [47], and it has been suggested that a higher dosing regimen may be necessary to achieve pharmacokinetic/pharmacodynamic targets [48].

The findings of our review have major implications for clinical practice. If CFDC or SUL/DUR do not outperform optimal high-dose-AMP/SUL-based regimens, there is little reason to routinely prefer these newer, significantly more expensive, and broader-spectrum options. In this scenario, CFDC or SUL/DUR would be reserved for selected cases. For instance, CFDC- or SUL/DUR-based regimens may be preferable for CRAB strains with high (> 32–64 mg/L [49]) SUL minimum inhibitory concentration (not accounting for the potential role of synergistic combinations in overcoming SUL resistance) or in case of intolerance to AMP/SUL. Additionally, for MBL-producing strains, where SUL/DUR is inactive [19], CFDC-based regimens may be a viable option [50]. However, there is concern that CFDC’s activity may be suboptimal against NDM-producing isolates [16, 17], in which cases CFDC-based synergistic combinations could be beneficial, pending approval of novel beta-lactamase inhibitors that are active against MBLs [51].

This review has some limitations. Given the recent approval of SUL/DUR, we did not find any eligible published studies beyond the registrational Phase 3 [20]. Importantly, SUL/DUR was combined with imipenem in this trial, and while there are valid mechanistic explanations for potential synergy [52, 53], there is no clinical evidence confirming improved outcomes by the combination. Furthermore, as per IDSA guidance [14], we defined BAT as a regimen based on high-dose-AMP/SUL, but strong supportive evidence is lacking [5, 6, 14]. Additionally, the best adjunctive antimicrobial to add to high-dose AMP/SUL remains uncertain, with IDSA suggesting multiple options (polymyxin B/ CST, minocycline/ tigecycline, CFDC) [14]. Recent data suggest that fosfomycin, as part of synergistic combinations, may improve outcomes [9, 24], even though IDSA advises against routine use [14]. Lastly, a key limitation in evaluating treatment options for CRAB is the problematic in vitro susceptibility testing of CFDC [54], the absence of established breakpoints for several key antibiotics (e.g. lack of EUCAST breakpoints are lacking for CFDC, AMP/SUL, tigecycline and minocycline [55]) and the limited data correlating in vitro activity and resistance mechanisms with clinical outcomes.

In conclusion, available data have serious limitations, and there is a lack of high-quality evidence supporting routine use of either SUL/DUR or CFDC (both substantially more expensive than alternative BAT) over high-dose AMP/SUL-based regimens for unselected patients with CRAB infections. Trials directly comparing the various options are necessary to define the optimal therapy for infections by CRAB, considering contemporary BAT, antimicrobial stewardship, and financial burden of newer agents.

## Data Availability

No datasets were generated or analysed during the current study.
